# Orexin-A Modulates Firing of Rat Rostral Ventromedial Medulla Neurons: An *In Vitro* Study

**DOI:** 10.22074/cellj.2015.524

**Published:** 2015-04-08

**Authors:** Hassan Azhdari-Zarmehri, Saeed Semnanian, Yaghoub Fathollahi

**Affiliations:** 1 Department of Physiology, School of Medical Sciences, Tarbiat Modares University, Tehran, Iran; 2 Department of Basic Sciences, Torbat Heydariyeh University of Medical Sciences, Torbat Heydariyeh, Iran

**Keywords:** Orexin-A, SB-334867, Patch Clamp, Rat

## Abstract

The rostral ventromedial medulla (RVM) acts a key role in the descending inhibitory pain modulation. Neuropeptide orexin-A (ORXA) is confined to thousands of neurons in the lateral hypothalamus (LH). While RVM gets the orexinergic projections, the orexin receptors are also expressed in this structure. The aim of this study was to specify the cellular effects of ORXA on RVM neurons *in vitro* by using the whole cell patch-clamp recording. RVM neurons were classified into three types based on their electrophysiological characteristics. Type 1 neurons exhibited an irregular spontaneous activity which was interrupted by periods of pause in 25% of recorded neurons. Type 2 neurons did not show any spontaneous baseline activity (53.8% of recorded neurons). Type 3 neurons fired repetitively without interruption (51.2% of recorded neurons). ORXA had either inhibitory or excitatory effects on 53.8% (7/13) of type 1 neurons. ORXA excited 46.4% (13/28) of type 2 neurons and 27.3% (3/11) of type 3 neurons. The excitatory effect of ORXA observed in type 2 neurons was suppressed by an orexin 1 receptor (OXR1) antagonist, SB-334867. Briefly, we hypothesized that the ORXA mediated excitation and/or inhibition in RVM neurons might work as a mechanism to modulate pain processing by orexinergic neurons.

Rostral ventromedial medulla (RVM) with oncell and off-cell type neurons participates in the descending control of up-down pain modulation through the first synapse in the dorsal horn of the spinal cord and is a major site ofanalgesic actions of opioids (1-3). A simultaneous extracellular single unit recording along with a noxious stimulus applied to either the tail or paw has shown different types of neurons: onand off-cells are discriminated by a sudden burst and a sudden pause in firing rate, respectively, in which both cell types associate with nociceptive reflexes (4, 5). On-cells are directly inhibited with opioids such as morphine and in many studies it has been reported that these neurons can facilitate nociception (4, 6).

The neuropeptides orexin-A (ORXA) and -B (ORXB) are mostly expressed in neurons of the lateral hypothalamus (LH) (7). Sakurai et al. (7) havedescribed two types oforexin receptors coupled to G proteins. Orexin is implicated in the regulation of various brain and body functions such asfeeding (8, 9), sleep (10, 11), locomotion (12), reward and addiction (13-15). Recently, a wealth of experimental evidence shows a role for orexin in pain control (16-24). Morphological studies have established that orexin projections as well as orexin receptors are distributed along most centers that participate in pain processing including the RVM region which is considered to be important for pain modulation (25-27).

In our previous behavioral study we usedthe formalin test and reported a decrease in formalin induced nociceptive behaviors after ORXA microinjection into the RVM that was blocked by an orexin-1 receptor (OXR1) antagonist, SB-334867 (28). Furthermore, we showed that intracerebroventricular (ICV) injection of ORXA inhibited or decreased the spontaneous firing rate of on-cells [the type of RVM neurons believed to have facilitatory action on nociception) and increased the firing rate of off-cells (the type of RVM neurons believed to have an inhibitory action on nociception]. RVM might directly or/and indirectly [projections from periaqueductal gray (PAG)] serve as an important site of action for orexin-induced supraspinalantinociception. Therefore, the present study was conducted to examine the *in vitro* effects of ORXA on RVM neurons using the whole cell patch-clamp recording technique. These data were presented as an abstract at Neuro 2010 in Japan (29).

Adult Sprague-Dawley rats were purchased from Razi Institute (Karaj, Iran). Animals were housed at a temperature of 22 ± 2˚C under a 12 hour light-dark cycle with lights on from 7:00 to 19:00. Food and water were freely provided. All experiments involving the animals were conducted according to the protocols approved by the Ethics Committee of Tarbiat Modares University (TMU), Tehran, Iran. In all experiments, attention was paid to decrease affliction during low-stress handling with animals.

After brief anesthesia with ether, 12 to 16-day-old pups were decapitated and the brains immersedin cold artificial cerebrospinal fluid (ACSF) composed of 125 mM NaCl, 3 mM KCl, 0.1 mM CaCl_2_, 5 mM MgCl_2_, 1.25 mM NaH_2_PO_4_, 10 mM D-glucose, 0.4 mM L-ascorbic acid and 25 mM NaHCO_3_ (Sigma-Aldrich), pH=7.4 bubbled with 95% O_2_+5% CO_2_; osmolarity: 310 m Osm/kg). The cerebellum was removed from each brain and the brain stem block was affixedby cyanoacrylate glue to a slicing tray filled withice-cold ACSF in which the concentration of MgCl_2_ was increased to 5 mM while that of CaCl_2_ decreased to 1 mM. Coronal slicesof 300-400 μm thicknesses were cut through the brain stem from the trapezoid body to the inferior olivary nuclei using a vibrating microtome (Vibatome 1000 plus, USA). The slices were first incubated in a holding chamber at a constant flow of standard ACSF with 2 mM CaCl_2_ and 1.3 mM MgCl_2_ at 35-37˚C for 30-45 minutes. The slices were kept at room temperature (20-25˚C) in the same chamber until electrophysiological recording. The slices were super fused at a rate of 1-2 ml/min with standard ACSF (30).

Neurons in the RVM were visualized in the triangular midline region dorsal to the pyramidal tracts (31, 32) and visually identified under an upright microscope (Axioskop, Zeiss, Germany) with infrared differential interference contrast (IR-DIC) optics. The IR-DIC images were captured using a CCD camera (DAGE-MTI, Germany) and digitally stored on a computer. Patch pipettes with a resistance of 3-6 Mohms were made from hard borosilicate glass using a pipette puller (P-97, Sutter, USA). Holding potentials, data acquisition and analysis were controlled by an on-line personal computer programmed with pCLAMP 10 (Axon Instruments). Current and voltage signals obtained by a patch-clamp amplifier (Axopatch-700B, Axon Instruments, USA) were sampled at 10 kHz (Digidata 700B interface, Axon Instruments, USA) and stored on the hard disk of a computer for off-line analysis with Clamp fit 10.

We used pCLAMP to measure the passive membrane properties in voltage-clamp modeaccording to a study by Zhang et al. (33) Liquid junction potentials were 10 mV as calculated with Clampex 10 (Axon Instruments, USA) for our internal solutions, which was not corrected.

One wayanalysis of variance (ANOVA) was used to compare the passive membrane and action potential properties of three different populations of neurons. Dunn’s test was used for post hoc comparisonsamong mean values for the individual groups. P<0.05 was considered significant.

According to Zhang et al. (33) three classes of RVM cells couldbe discriminated on the basis of their electrophysiological activities. Type 1 neurons showed spontaneous activity which stopped with periodsof pause in the spontaneous activity (Fig.1A, B). Type 2 neurons were spontaneously inactive (Fig.1C, D). Type 3 neurons were spontaneouslyactive and fired repetitively (Fig.1E, F). All threetypes could be subdivided into two groups: neurons without fast after hyperpolarization potential (AHP) as seen in figure 1A, C, E where one action potential extended into the left and top of this figure. Neurons with fast AHP are visualized in figure 1B, D, F. The entire cell patch clamp recordings were conducted from a total of 52 RVM neuronson the current clamp mode (type 1: 28.1% (9/32) and 20 % (4/20), p=0.1 with or without fast AHP, respectively; type 2: 50% (16/32) and 60% (12/20), p=0.29; type 3: 21.8% (7/32) and 20% (4/20), p=0.86, with or without fast AHP, respectively, table 1).

The action potential and passive membrane properties of three classes of cells thatwere recorded in RVM are compared in table 1.

**Fig.1:**
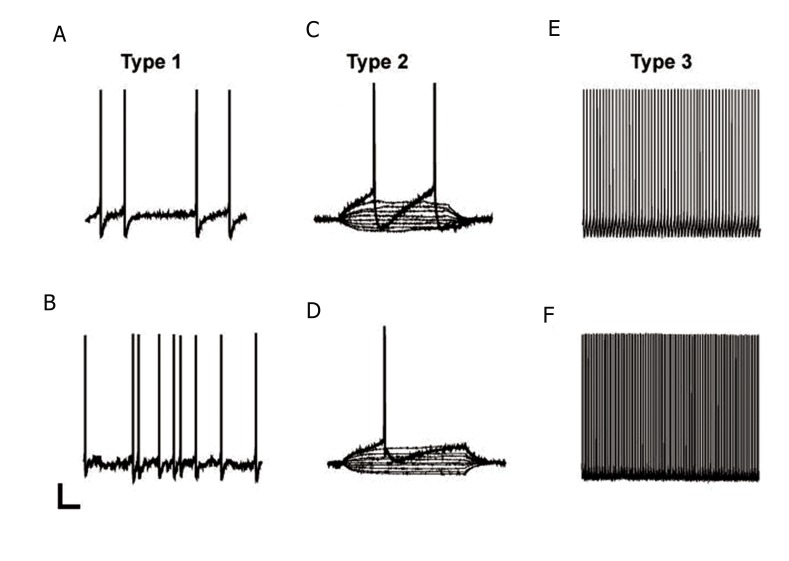
Representative examples of type 1 (A, B), type 2 (C, D) and type 3 (E, F) rostral ventromedial medulla (RVM) neurons. A, C and E. Neurons with fast after hyperpolarization potential (AHP). B, D and F. Neurons without fast AHP. In type 2 neurons, which exhibited no spontaneous activity, the action potentials were evoked by injection of a depolarizing current (scale bar for A, B, E and F=20 mV and 1 s. Scale bar for C and D=20 mV and 100 ms).

**Table 1:** Passive membrane and action potential properties in RVM neurons with and without AHP


Parameters	Type 1		Type 2		Type 3	
	Without fast AHP	With fast AHP	Without fast AHP	With fast AHP	Without fast AHP	With fast AHP

**Spontaneous activity**	9/9	4/4	0/16	0/12	7/7	4/4
**Action potential threshold(mv)**	-34.88 ± 0.54	-36.82 ± 0.04	-38.39 ± 1.19	-38.32 ± 1.82	-36.22 ± 2.57	-35.57 ± 1.2
**Action potential amplitudefrom threshold (mv)**	77.2 ± 3.05	75.7 ± 5.67	79.2 ± 2.55	76.4 ± 2.35	69.3 ± 4.24	72.8 ± 4.16
**Time to peak of actionpotential (ms)**	0.54 ± 0.08	0.50 ± 0.07	0.73 ± 0.10	0.49 ± 0.06	0.86 ± 0.13	0.96 ± 0.23
**Action potential thresholdto AHP maximum (mv)**	34.31 ± 0.34	34.5 ± 0.35	18.7 ± 3.15	21.9 ± 4.17	21.8 ± 3.07	28.1 ± 7.09
**Time to peak of AHPmaximum (mv)**	1.33 ± 0.16	1.0 ± 0.2	2.0 ± 0.35	1.4 ± 0.15	1.9 ± 0.20	2.5 ± 0.68
**Action potentialhalf-width (ms)**	1.57 ± 0.21	1.72 ± 0.01	1.61 ± 0.20	1.16 ± 0.10	1.97 ± 0.16	2.06 ± 0.35
**Membrane resistance(MΩ)**	420.0 ± 176.2	366.7 ± 109.2	590.6 ± 118.2	279.5 ± 59.3	221.3 ± 21.5	221.3 ± 21.5
**Resting membranepotential (mv)**	-50.1 ± 1.79	-48.8 ± 3.38	-60.3 ± 2.10	-58.5 ± 1.62	NA	NA


Values are means ± standard error (SE). NA; Not applicable, RVM; Rostral ventromedial medulla and AHP; After hyperpolarization potential.

Current-clamp recordings from 52 RVM cells showed that 48% (24 out of 52) of this population responded to bath perfusion of ORXA whereas the remainder of neurons was classified as non-responders (NON). In type 1 without fast AHP, inhibitionwas the predominant effect caused by ORXA exposure at the 100 nM concentration (Fig.2A) as seen in 5 of 9 (50.5%) cells. In type 1 with fast AHP, excitationwas the predominant effect caused by ORXA exposure at the 100 nM concentration (Fig.2B) in 2 of 4 (50%) cells. In type 2 with and without fast AHP, excitationwas the principal effect caused by ORXA exposure at the 100 nM concentration (Fig.3A, B) in 9 of 16 (66.6%) cells and 4 of 12 (33.3%) cells. In type 2 with fast AHP, 1 neuron was inhibited after administration of 100 nM ORXA (data not shown). Type 2 neurons were characterized as those that lack spontaneous activity, thus it wasdifficult to visualize inhibition when the cell was not active and observation of excitatory effects of orexin was prevalent. In type 3 with and without fast AHP, excitationwas the predominant effect caused by ORXA exposure at a nanomolar concentration (Fig.4) in 2 of 7 (28.5%) cells and in 1 of 4 (25%) cells, respectively. However, the excitatory effect of ORXA on type 2 neurons of RVM was blocked by SB-334867 (10 μM) which demonstrated OXR1 involvement (Fig.5).

**Fig.2:**
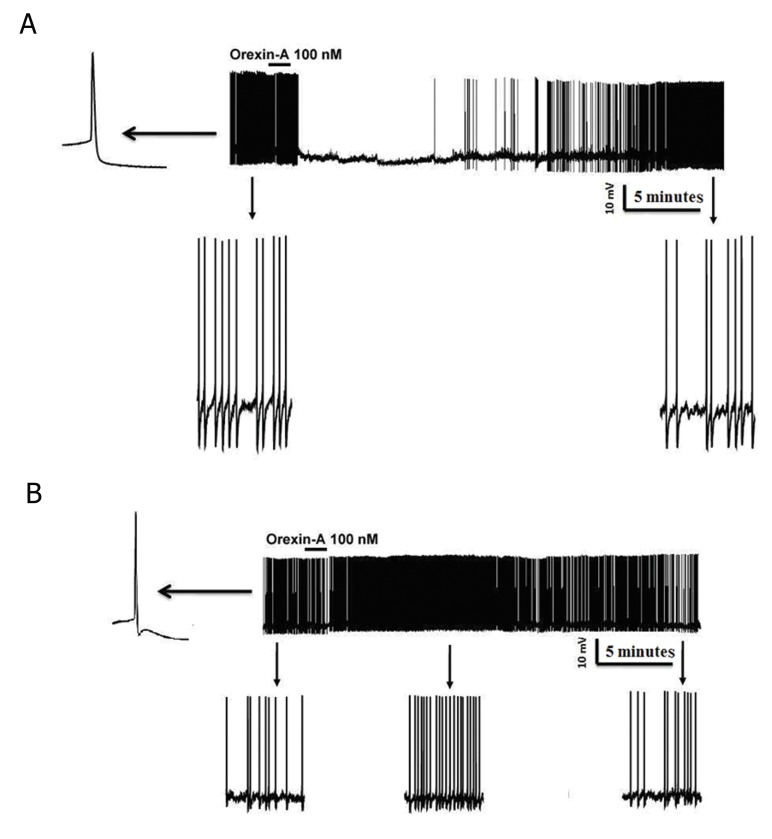
Whole cell patch recording of rostral ventromedial medulla (RVM) type 1 neurons in a slice under the current clamp. In type 1 cells without fast after hyperpolarization potential (AHP) (A), bath application of orexin-A (ORXA) resulted in hyperpolarization and inhibited the firing rate of the neurons. In those with fast AHP (B), ORXA resulted in depolarization and increased frequency of action potentials. This effect was reversed after a washout period. The bar indicates the presence of orexin in the recording chamber. Bottom of A and B: expanded time scales from the same recording (before and after bath application of ORXA).

**Fig.3:**
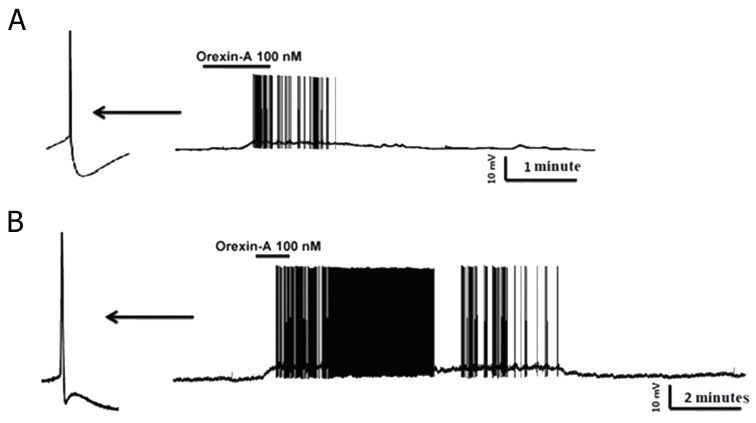
A. Whole cell current-clamp recording from a rostral ventromedial medulla (RVM) type 2 neuron which demonstrated that bath administration of orexin-A (ORXA) resulted in rapid sustained depolarization accompanied in most cases by a rapid increase in firing frequency of action potentials in both neurons without fast after hyperpolarization potential (AHP) and B. with fast AHP. After washout of ORXA, the membrane potential and action potential rate returned to control levels. Bottom of A and B. expanded time scales from the same recording (before and after bath application of ORXA).

**Fig.4:**
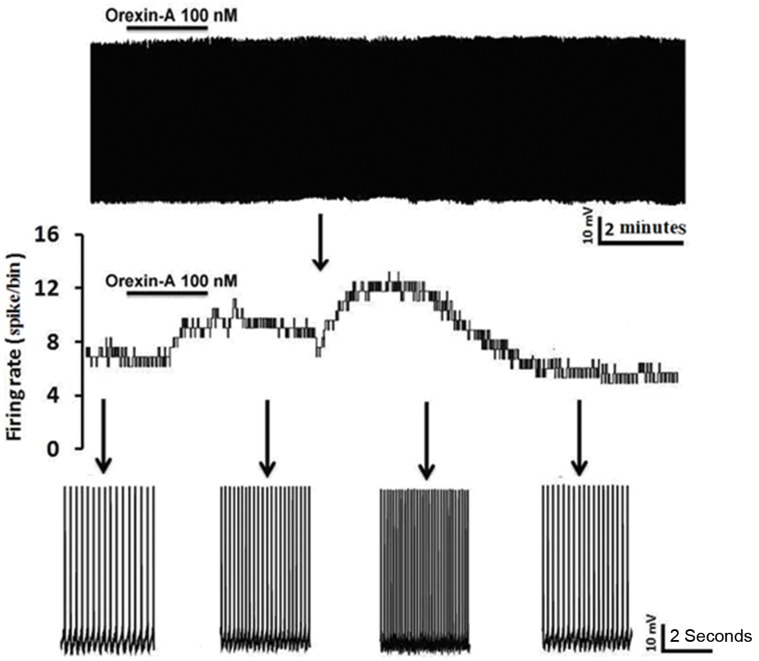
Whole cell current-clamp recording from an rostral ventromedial medulla (RVM) type 3 neuron demonstrating a rapid increase in firing frequency of action potentials following bath administration of orexin-A (ORXA). After washout of ORXA, the action potential frequency returned to control levels. Bottom: expanded time scales from the same recording (before, after bath application and during recovery of the ORXA effect).

**Fig.5:**
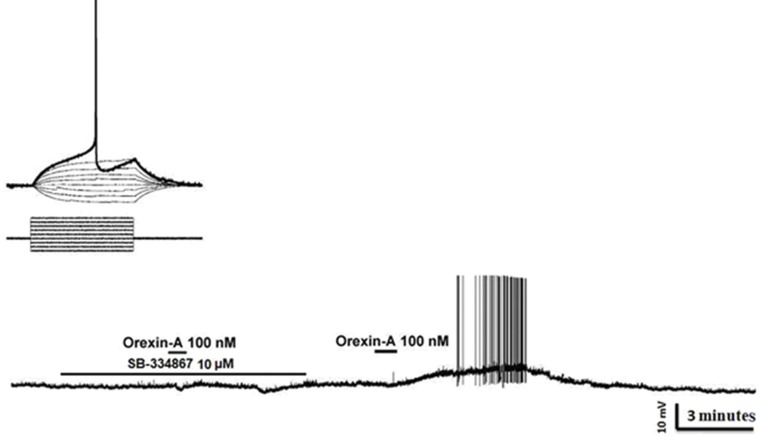
Characterization of orexin receptor type 1 (OXR1) involved in depolarizing responses of type 2 cells. Above: Current injection induced action potential (each step is 30 pA). Bottom: SB-334867 (OXR1 antagonist, 10 μM) completely blocked the orexin-A (ORXA)-induced depolarization. ORXA (100 nM) depolarized and induced action potential after washout of SB-334867.

In this study, we classified RVM neurons into three types based on their electrophysiological characteristics. Type1 neurons exhibited an irregular firing activity which was inactivated by periods of pause (25% of decoded neurons), is similar with secondary cell type which described by Pan et al. (31, 32). Type 2 neurons did not show any spontaneous basal activity that might correspond with the primary cell in their studies type 3 neurons fired continuously without pause in 51.2% of decoded neurons. This classification was performed according to previous studies. In another complementary study by Zhang et al. (33) which concerned analysis of passive membrane properties and action potential characteristics of spinally projecting RVM neurons, the authors concluded that RVM neurons could be distinguished from their highly characteristic pattern of spontaneous activity.

ORXA modulation of RVM neuronal activity was as follows. Only 48% of RVM neurons (24 of 50) were modulated by ORXA, with12% hyperpolarized and 36% depolarized.These results agreed with immunocytochemical observations which indicated that a few RVM cells were OXR1 immunoreactive (34). The inhibitory effect of ORXA on type 1 cells was consistent with hyperpolarization effects of opioids in the secondary cell in study of Pan et al. (31), which suggested an *in vivo* correlation with on-cells. The excitatory effect of ORXA on type 2 cells was consistent with actions of opioids in primary cellsin study of Pan et al. (31) suggested a correlation with off-cells *in vivo*. On the other hand, the modulatory effects of ORXA on RVM neurons were consistent with the fact that ORXA has been shown to exert two opposite effects on two distinct sets of mitral cells: either a direct depolarizing effect, or an indirect hyperpolarizing effect (35). The effects of ORXA on mitral cells were blocked by SB-334867-A, which showed the involvement of OXR1 (35). Based on the effect of ORXA on mitral cells, it was possible that the differential effects could be the result of postsynaptic (direct) and presynaptic (indirect) actions.To understand the mechanism of ORXA effect on RVM neurons, additional studies should be conducted in the presence of tetrodotoxin to ascertain whether the excitatory and inhibitory actions in type 1 neurons are direct or indirect.

Our electrophysiologicalresults were consistent with our *in vivo* study (36). Applications of ORXA were shown to induce a decrease in spontaneous rate of on-cells that were likely involved in descending facilitation (37). The selective orexin receptor antagonist, SB-334867, has been shown to display a 20-fold higher affinity for OXR1 than OXR2 (37). SB-334867 blocked the excitatory effect of ORXA on type 2 neurons of RVM,which emphasized the involvement of OXR1.

Recently Zhang et al. (33) used the whole cellpatch-clamp recording technique and have reported the existence ofthree heterogeneous types of cells in RVM. Type1 neurons exhibited irregular spontaneous basal activity. Type 2 neurons werenot active spontaneously. Type 3 neurons fired repetitively withoutinactivation periods. Zhang et al. (33) reported that serotonergic neurons had a higher membrane resistanceand greater action potential than their non-serotonergicones and did not exhibit a fast after-hyperpolarization which was consistent with this study.

As ORXA is involved in different states and functions, the present results provide new, important electrophysiogical evidence that ORXA-modulated RVM neurons in a heterogeneous manner may be involved pain modulation.
